# Insight of Ethnomedicines in Dentistry: A Brief Review

**DOI:** 10.7759/cureus.28148

**Published:** 2022-08-18

**Authors:** Aishwarya A Gupta, Anendd Jadhav, Nitin Bhola, Pooja Agrawal

**Affiliations:** 1 Oral and Maxillofacial Surgery, Sharad Pawar Dental College and Hospital, Datta Meghe Institute of Medical Sciences, Wardha, IND

**Keywords:** herbal mouthwashes, therapeutic herbs, analgesics, anti-inflammatory, ethnomedicine

## Abstract

Ethnomedicines in the literature compare the therapeutic efficacy of various herbs based on active ingredients of plants and animals. The application of phytomedicines in the field of dentistry is uncommon. The main objective of this article is to access the efficacy of ethnomedicines and newly evolving treatment modalities in reducing post-op complications following dentoalveolar surgeries. Inclusion criteria were selected according to the population, intervention, control, and outcomes (PICO) format. Case reports, case series, retrospective studies, and studies with inappropriate reporting of outcomes were all excluded. An electronic search of English literature in PubMed was performed using the keywords Ethnomedicine, Anti-inflammatory, Analgesics, Therapeutic herbs, Herbal mouthwashes, Third molar surgery. A total of 25 articles were selected, of which three were on herbal mouthwashes and 22 were on anti-inflammatory effect. All the articles were regarding the therapeutic effect of the herbs. The present paper studies various traditionally used therapeutic herbs, their benefits, and shortcomings with their application in dentistry. This study has shown the different herbal alternatives to conventionally used drugs in relation to third molar.

## Introduction and background

Ethnomedicine is a study of evaluation of traditional medicine based on bioactive compounds in plants and animals. It is the area of anthropology that studies different societies; notations of health and illness constitute people’s thinking and action about well-being and healing [1]. They played a pivotal role in the invention of the new element from indigenous reported medicinal plants. Herbal extracts have historically proven to be beneficial in varied specialties and disciplines. The active ingredient in the herbs forms the molecular basis of newly developed pharmaceuticals. Ethnomedicine is pivotal in treating dental complications following minor surgical procedures with minimal side effects and drug interactions [2]. The natural source of drugs makes them readily acceptable to the general population.

Biomedicine is a branch of medical sciences that applies biological and other natural science principles to clinical practice, commonly popularised as allopathic drugs. It is considered the cornerstone of the modern era in health sciences. The conventionally used drugs by dental surgeons are non-steroidal anti-inflammatory drugs (NSAIDs) and steroids for treating postinflammatory complications following dentoalveolar surgery. The drugs' selective and rapid action has brought transformation in the field of medicine. On the contrary, the ferocity of the adverse effects has also increased with the increased contrivance in modern medicine. The search for new alternatives with minimal side effects is still burgeoning.

The surgical removal of the lower impacted third molar (LI3M) often results in significant postoperative discomfort, and its management demands relevant knowledge of the anatomy of contiguous structures. The common sequelae of LI3M surgery are pain, facial edema, and trismus arising secondary to tissue inflammation following surgical trauma to soft and bony tissue in the early postoperative period. These adversely influence the patient's quality of life by affecting overall comfort, limitation in daily routine activities, sleep, mastication, trismus, phonetics, and smiling [3].

To limit these postsurgical inflammatory complications, such as edema, pain, and trismus, firstly, surgeons have improvised surgical techniques and instrumentation for precise execution, and secondly, included various modalities like usage of NSAIDs, steroids, lasers, tapes, sutures, ozone gel, phyto-enzyme therapy, and cryotherapy. The present review aimed to explore the various perspectives of the holistic treatment approach in dentoalveolar surgery.

The current trend of prescription of analgesics is associated with the increasing prevalence of adverse effects. Ethnomedicine is a promising branch that deals with herbal extracts with minimal adverse effects. Hence, a mini-review is required to access the efficacy of these ethnomedicines and newly evolving treatment modalities. This mini-review analyzed various anti-inflammatory agents in ethnomedicines against the routinely prescribed biomedicines. This paper discussed the potential of phytomedicine in reducing the postoperative inflammatory sequelae following dentoalveolar surgery.

**Figure 1:**
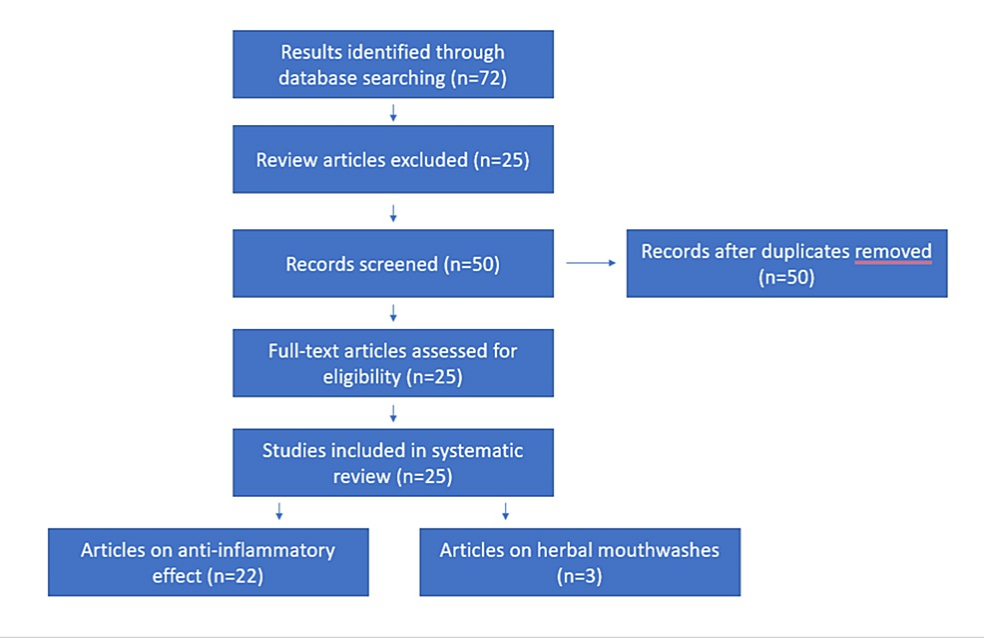
Preferred Reporting Items for Systematic reviews and Meta-Analysis (PRISMA) flow diagram

The research question was framed according to the Preferred Reporting Items for Systematic reviews and Meta-Analysis (PRISMA) guidelines (Figure 1). The research question was ‘Which are the herbal extracts possessing anti-inflammatory properties?’ since 2011. The inclusion and exclusion criteria are given in Table 1.

**Table 1:** Inclusion and Exclusion criteria PICO: population, intervention, control, and outcomes, NSAIDs: non-steroidal anti-inflammatory drugs

Inclusion Criteria- PICO format	Exclusion Criteria-
Participants ≥ 15 years of age.	Case reports
Intervention: Bromelain; Arnica Montana; Aloe-vera, Binahong, pisidium guajava, Curcumin, Calendula, Chamomile flower, Pomegranate, Hypericum perforatum, Green tea, Passiflora incarnata, Propolis.	Case series
Comparator: NSAIDs, Anti-bacterial mouthwashes- Chlorhexidine.	Retrospective studies
Outcomes: The primary outcome variables were the assessment of the herbal agents' anti-inflammatory, analgesic, anti-bacterial and anti-microbial properties. The secondary outcome evaluated the wound healing potential and prevalence of adverse effects with the use of these ethnomedicines.	Studies with inappropriate reporting of outcomes.

The search strategy was an electronic search of English literature in PubMed was performed using the keywords Ethnomedicine, Anti-inflammatory, Analgesics, Therapeutic herbs, and Herbal mouthwashes.

## Review

Bromelain is a phytoenzyme with proteolytic action extracted from the pineapple stem (Ananas comosus) in late 1957. Bromelain has gained attention in dentoalveolar surgery due to its anti-inflammatory, analgesic, anti-fibrinolytic, and antimetastatic properties [4,5]. Some studies postulated the mechanism of action of bromelain by affecting the concentration of cyclo-oxygenase two enzymes and thereby leukocyte activation. It acts as a bradykinin blocker and reduces prostaglandin resulting in anti-inflammatory activity. It is a drug with moderate efficacy and minimal adverse effect. Bromelain significantly reduces the postoperative inflammatory sequelae following removal of the impacted third molar [2]. Although bromelain causes fibrinolysis and restricts the blood coagulation cascade by affecting factor X, it also reduces ‘prekallikrein’ and thereby reduces bradykinin formation. Overall will lead to increased bleeding tendency and should be avoided in patients with bleeding disorders [6].

Along with the bromelain, other botanical extracts have also shown their efficacy in reducing inflammatory sequelae. Calendula, botanical antioxidants, contains many flavonoids, producing free radicals, effective against bacteria and viruses. It accelerates wound healing by enhancing vascularity in the maimed tissue [7]. The chamomile flower constitutes α-bisabolol oxides A & B and matricin.These are then converted to chamazulene and other flavonoids. It acts by inhibiting lipo-oxygenase and thereby reducing prostaglandins production. Also, it selectively works on COX-2 sparing COX-1, and exhibits anti-phlogistic properties [2]. Several authors have demonstrated the excellent wound healing potential of cinnamon [8].

Curcumin is an active ingredient found in various natural herbs. Turmeric is the derivative of Curcuma longa and belongs Zingiberaceae family. It has antioncogenic, antidiabetic, antihypertensive, anti-inflammatory, anti-psoriatic, and anti-microbial characteristics [9]. Multimodal studies have evaluated the anti-phlogistic efficacy of curcumin. It acts by inhibiting cyclooxygenase (COX)-2 and thereby causes deceleration in the production of IL-12, leukotrienes, prostaglandin E2 (PGE2), hyaluronidase, nitric oxide, lipoxygenase, tumour necrosis factor α (TNFα), monocyte chemoattractant protein 1 (MCP-1) and other inflammatory mediators. Satoskar et al. have demonstrated curcumin’s anti-inflammatory property on postoperative inflammation [9].

Binahong (Anredera cordifolia) constitutes flavonoids, terpenoids, oleanolic acid, ascorbic acid, tannins, and saponins. Flavonoids stifle the arachidonic acid pathway and thereby minimize the PGE2 production. Triterpenoids and saponins exhibit the anti-microbial action by interfering the cell membrane formation and increasing the cell wall permeability leading to the release of proteins and causing cell death. Saponins suppress the growth of microbes and exhibit anti-septic properties. Hanafiah et al. have demonstrated the dose-dependent action of binahong leaf extract in promoting mucosal wound healing [10,11].

Arnica is an active ingredient extracted from various phyto-derivatives belonging to the Asteraceae family, constituting Arnica montana, A. chamissonis, A. fulgens, and A. cordifolia. Multimodal studies have shown the efficacy of A. montana as an anti-phlogistic agent. It has shown significant effects in reducing inflammation and hematoma. Arnica has shown its efficacy in treating osteoarthritis, myocardiopathy, arteriosclerosis, sprains, edema, and other acute inflammatory conditions [12,13].

Pomegranate (Punica granatum) has shown anti-microbial action comparable to chlorhexidine in dentistry. Several authors evaluated the anti-inflammatory property of Punica granatum, and its mouthwash on microbes was recorded by Keville. It acts on the cyclo-oxygenase pathway, thereby leading to decreased production of prostaglandins. The pomegranate has shown excellent activity as an anti-inflammatory agent and fast recovery [14].

Propolis is one of the phyto-derivative used to relieve pain following dentoalveolar surgery by reducing inflammation. Magro Filho et al. [15] demonstrated topical propolis hydroalcoholic solution application's application to enhance epithelial repair in the post-extraction socket. Propolis has shown its efficacy in treating alveolar osteitis. Abdullah et al. [16] showed propolis extract’s effect in reducing the severity following third molar removal. Several studies have concluded the anti-phlogistic and anti-cariogenic potential of Brazilian propolis. It exhibits bactericidal, fungicidal, anti-inflammatory, and antioxidative properties by inhibiting COX-2 and minimizing PGE2 biosynthesis. It also acts by inhibiting nitric oxide and reducing the inflammatory mediators (IL-1𝛽, IL-2, IL-6, IL-10, and TGF-𝛽) and by causing degradation of free radicals produced by macrophages and poly-morpho neutrophils. It promotes granulation tissue formation and thereby aids in wound healing [15,16].

Passiflora incarnata, one of the traditional herbal derivatives belonging to the Passifloraceae family, is commonly used as an anxiolytic agent. Multitude studies have demonstrated the anxiolytic action of Passiflora without inducing sedation in patients undergoing dentoalveolar surgery. It increases the gamma-aminobutyric acid level and lowers neuronal activity, causing sleep. Another Brazilian herb, Erythrina mulungu, is an analgesic, anxiolytic, anticonvulsant, hypnotic, sedative, and hypotensive drug. It has been successfully used in patients undergoing removal of the impacted lower third molar. Studies have assessed the efficacy of Erythrina mulungu, Passiflora incarnata, and midazolam as anxiolytic agents in patients undergoing third molar surgery and demonstrated comparable efficacy of the phyto-derivatives with midazolam as an anxiolytic drug [17,18]. Ambient orange can also be used as an anxiolytic agent during minor surgical procedures [19].

Hypericum perforatum is an ancient European therapeutic herb that constitutes phloroglucinols, naphthodianthrones, xanthates, flavanol, and phenolic derivatives. It is commonly popularised as St. John’s wort formulation. Hypericin and hyperforin exhibit their anti-inflammatory, anti-oncogenic potential. It acts by impeding the production of IL-12. Hyperforin, a naphthodianthrone, acts by halting COX-1 and lipo-oxygenase. Multiple studies have shown the anti-phlogistic properties of hypericum oil with accelerated wound healing [20,21].

Green tea is a widely considered rejuvenating potion among the general population. Catechins constituted in the green tea formulation (Epigallocatechin (EGC), Epicatechin (EC), and Epigallocatechin-3-gallate (EGCG)) exhibit anti-bacterial and analgesic properties. The efficacy of green tea is comparable with chlorhexidine mouthwash and low-level laser therapy (LLLT). Multitude studies have demonstrated significant analgesic properties with moderate antibiotic action and minimal side effects in treating postoperative complications following dentoalveolar surgeries [22,23].

Ipomoea batatas (sweet potato) is one of the most commonly consumed herbs among East Asians belonging to the Convolvulaceae family. Several authors have assessed its anti-phlogistic, anti-asthmatic, anti-oxidant, anti-convalescent, antitumoral, aphrodisiac, and antidiabetic action. It acts by fettering several metabolic pathways causing inflammatory mediators' production, ultimately halting the production of PGE2, leukotrienes, and cytokines. It also works by inhibiting active nitrogen and oxygen species, commonly known as anti-oxidant agents. Many studies have demonstrated the anti-microbial action of Ipomoea batatas and its action on Streptococcus and Staphylococcus species. Also, this therapeutic herb has shown moderate efficacy in reducing inflammation following dentoalveolar surgeries [24].

Pisidium guajava (guava) is the most popularised fruit among South Asians. Its leafy extract exhibits therapeutic action in treating gastrointestinal disorders and respiratory diseases. The leaves of Pisidium are commonly used in Western countries as an antibiotic in the form of poultice and decoction for treating inflammatory ulcers. Several authors have studied the anti-inflammatory, anti-cestode, analgesics, anti-microbial and antioxidant properties of Pisidium. It acts by impeding the production of histamine, kinins, and prostaglandins. Current studies have assessed Pisidium extract's application in dentistry and shown moderate efficacy in treating inflammatory complications following minor dental procedures [1].

Aloe barbadense (aloe vera) is popularised among the general population due to its therapeutic actions. It acts by inhibiting COX-2 and the production of PGE2 and other inflammatory mediators like cytokines and leukotrienes. Aloe barbadense constitutes a brady kinase enzyme leading to the breakdown of bradykinin which is a potent mediator of pain. It has shown action on granulation tissue formation by accelerating the cross-linking among the aldehyde content. The hormonal content of aloe vera constitutes auxins, and gibberellins are responsible for the anti-phlogistic action of the herb. More than a few studies have demonstrated aloe vera's antiphlogistic, analgesic, and anti-bacterial properties. Recent literature highlighted the comparable anti-bacterial efficacy of aloe vera mouthwashes to chlorhexidine mouthwash following third molar surgeries [25,26].

## Conclusions

This systematic review has included studies on the therapeutic action of the herbs from 2011 to 2022. It has not comprised all the parameters of post-op complications following dentoalveolar surgeries. The present paper reviewed various botanical extracts aiding in healing and reducing post-op complications following dentoalveolar surgeries. The study may have the potential to outweigh the conventional practice. This paper emphasizes new alternatives to conventionally used biomedicines with minimal side effects.
